# *ggcoverage*: an R package to visualize and annotate genome coverage for various NGS data

**DOI:** 10.1186/s12859-023-05438-2

**Published:** 2023-08-09

**Authors:** Yabing Song, Jianbin Wang

**Affiliations:** School of Life Sciences, Tsinghua University, Beijing, China

**Keywords:** Genome coverage, Genome annotation, Visualization, Next-generation sequencing, Multi-omics

## Abstract

**Background:**

Visualizing genome coverage is of vital importance to inspect and interpret various next-generation sequencing (NGS) data. Besides genome coverage, genome annotations are also crucial in the visualization. While different NGS data require different annotations, how to visualize genome coverage and add the annotations appropriately and conveniently is challenging. Many tools have been developed to address this issue. However, existing tools are often inflexible, complicated, lack necessary preprocessing steps and annotations, and the figures generated support limited customization.

**Results:**

Here, we introduce *ggcoverage*, an R package to visualize and annotate genome coverage of multi-groups and multi-omics. The input files for *ggcoverage* can be in BAM, BigWig, BedGraph and TSV formats. For better usability, *ggcoverage* provides reliable and efficient ways to perform read normalization, consensus peaks generation and track data loading with state-of-the-art tools. *ggcoverage* provides various available annotations to adapt to different NGS data (e.g. WGS/WES, RNA-seq, ChIP-seq) and all the available annotations can be easily superimposed with ‘ + ’. *ggcoverage* can generate publication-quality plots and users can customize the plots with *ggplot2*. In addition, *ggcoverage* supports the visualization and annotation of protein coverage.

**Conclusions:**

*ggcoverage* provides a flexible, programmable, efficient and user-friendly way to visualize and annotate genome coverage of multi-groups and multi-omics. The *ggcoverage* package is available at https://github.com/showteeth/ggcoverage under the MIT license, and the vignettes are available at https://showteeth.github.io/ggcoverage/.

**Supplementary Information:**

The online version contains supplementary material available at 10.1186/s12859-023-05438-2.

## Background

Visualizing genome coverage is of vital importance to inspect and interpret various next-generation sequencing (NGS) data. Besides genome coverage, genome annotations are also crucial in the visualization. When analyzing whole-genome sequencing (WGS) data to get copy number variations (CNV), genome coverage plot can check for possible confounding factors, such as GC content bias, telomeres and centromeres proximity [1]. When dealing with RNA-sequencing (RNA-seq) data, we can utilize genome coverage plot to inspect the gene or exon knockout efficiency, 5’ or 3’ bias and visualize the read counts of differentially expressed genes, transcripts or exons [2]. In processing chromatin immunoprecipitation followed by sequencing (ChIP-seq) data, genome coverage plot can help to obtain and verify the peaks by comparing the signal of ChIP and input samples and visualize the relative distance between identified peaks and nearby genes [3].

Many tools have been developed to visualize genome coverage. However, existing tools are often inflexible, complicated, lack necessary preprocessing steps and annotations, and the figures generated support limited customization. For example, *UCSC genome browser* [4] and *IGV Browser* [5] require file upload or data transmission, which usually takes time, and are not accessible programmatically. *Gviz* [6] offers limited customization of plot aesthetics and themes. *ggbio *[7] and *GenVisR* [8] provide limited annotations. *karyoploteR* [9] focuses on visualizing chromosome ideogram and is complicated to create genome coverage plot. See Table 1 for a detailed comparison of *ggcoverage* to other visualization tools.

**Table 1:** Comparison of *ggcoverage* to other visualization tools

	*ggcoverage*	*ggbio*	*Gviz*	*GenVisR*	*karyoploteR*	*UCSC*	*IGV*
Platform	R	R	R	R	R	Web	Application
Data transmission						✓	✓
*Input file formats*							
BAM	✓	✓	✓		✓	✓	✓
BigWig	✓		✓		✓	✓	✓
BedGraph	✓		✓			✓	✓
TSV/Excel	✓					✓	
Sample group	✓		✓	✓	✓	✓	✓
*Preprocessing*							
Normalize reads	✓						
Consensus peak	✓						
Track loading (region subset)	✓	✓	✓		✓		
Protein coverage	✓						
*Annotations*							
GC content	✓		✓	✓	✓	✓	*
Base	✓	✓	✓		✓	✓	✓
Base frequency	✓				✓		✓
Amino acid	✓					✓	✓
CNV	✓			✓			
Gene	✓	✓	✓	✓	✓	✓	✓
Transcript	✓	✓	✓	✓	✓	✓	✓
Peak	✓		✓			✓	✓
Ideogram	✓	✓	✓		✓	✓	✓
Contact map	✓						
Link	✓				✓		
Feature	✓						
Superimpose annotations (‘+’)	✓	✓					
Customize figure (*ggplot2*)	✓	✓					

*IGV can load GC content from server, but has limited supported species

Here, we present *ggcoverage*, an R package providing a flexible, programmable, efficient and user-friendly way to visualize genome coverage of multi-groups and multi-omics. It supports multiple input file formats and provides several functions to perform data preprocessing, including parallel read normalization, consensus peak generation and track data loading. It also provides various available annotations, which can be superimposed conveniently to better inspect and interpret different NGS data. Furthermore, *ggcoverage* can generate publication-ready plots and users can customize the plots with *ggplot2* [10]. In addition, *ggcoverage* supports the visualization of protein coverage based on peptides obtained by mass spectrometry and adds protein feature annotation to the coverage plot.

## Implementation

### Inputs

The input file for *ggcoverage* to visualize genome coverage can be in BAM, BigWig, BedGraph and TSV formats. For TSV file, it should contain columns to specify chromosome, start, end, sample type and sample group. *ggcoverage* also requires additional files to generate annotation, such as FASTA file for GC content annotation, gene transfer format (GTF) file for gene and transcript annotations, and peak file for peak annotation. For the visualization of protein coverage, the input file should be Excel spreadsheets exported from an analyzer such as *Proteome Discoverer*.

### Data preprocessing

Read normalization, consensus peak generation and track data loading are usually prerequisites for visualization. However, this requires users to learn to use different tools and possibly switch between different platforms. To facilitate users, *ggcoverage* provides functions to perform data preprocessing with state-of-the-art tools. For read normalization, *ggcoverage* provides multiple normalization methods to adapt to different NGS data using *deeptools* [11] and parallelize this process with *BiocParallel* [12]. When providing peak files from replicates, *ggcoverage* can generate consensus peaks with *MSPC*, which can run with more than two replicates and combine evidence (e.g. P-value) from multiple replicates to obtain more reliable peaks [13]. To load track data, *ggcoverage* extracts the visualized region specified by users instead of loading the whole files and then extracting the visualized region.

### Visualization

*ggcoverage* introduces twelve layers to visualize and annotate coverage plot (Table 2). Besides these layers, *ggcoverage* also provides corresponding themes to beautify figures. geom_coverage will generate coverage plot of a specified region for different samples across different groups and provide ‘joint’ and ‘facet’ display styles (Additional file 1: Fig. S1). When mark regions are available, geom_coverage will also highlight these regions. geom_base is used to show base frequency and reference base for each locus, and it will also show amino acids of given region in *IGV* style. When SNVs exist, geom_base will highlight them with three styles (Additional file 1: Fig. S2). geom_cnv will show the normalized bin count and estimated copy number. geom_gc will calculate and visualize GC content of every bin, and it will also add a line to indicate mean GC content or user-specified GC content. geom_gene will obtain all genes in given region and classify these genes to different groups to avoid overlap when plotting. In gene annotation, the arrow direction indicates the strand of genes, the height of different elements indicates different gene parts, the color of line indicates gene strand or user-specified group information (e.g. gene type). geom_transcript is similar to geom_gene, but it shows all transcripts of a gene rather than the whole gene structure. geom_peak will show the peaks identified, so that the peaks and the nearby genes can be well visualized. geom_ideogram will show chromosome ideogram to illustrate the relative position of the displayed regions on the chromosome based on *ggbio* [7]. geom_tad will show 3D chromatin contact maps based on *HiCBricks* [14]. geom_link will create links of peak-gene or DNA-DNA. geom_protein will generate coverage plot of protein based on peptides obtained by mass spectrometry (Additional file 1: Fig. S3). geom_feature will show characteristics of protein or genome (Additional file 1: Fig. S3).

**Table 2:** *ggcoverage* layers

Layer	Type	Description
geom_coverage	Coverage	Create genome coverage plot
geom_base	Annotation	Add base, base frequency and amino acid annotations
geom_cnv	Annotation	Add CNV annotation
geom_gc	Annotation	Add GC content annotation
geom_gene	Annotation	Add gene annotation
geom_transcript	Annotation	Add gene’s transcripts annotation
geom_peak	Annotation	Add peak annotation
geom_ideogram	Annotation	Add chromosome ideogram annotation
geom_tad	Annotation	Add contact map annotation
geom_link	Annotation	Add link annotation
geom_protein	Coverage	Create protein coverage plot
geom_feature	Annotation	Add protein/genome feature annotation

Similar to graphical language implemented in *ggplot2*, users can superimpose the above layers by the ‘ + ’ operator with the help of *patchwork* [15]. For example, ggcoverage() + geom_gc() + geom_gene() + geom_ideogram() will create genome coverage plot and add GC content, gene structure and chromosome ideogram annotations.

### Customization

*ggcoverage* is based on *ggplot2*, so users can easily customize the generated figures with *ggplot2*. In general, customization mainly includes modifying the elements of existing figures and adding new layers. Additional file 1: Fig. S4 shows the examples of these two kinds of customization.

## Results

**Fig. 1:**
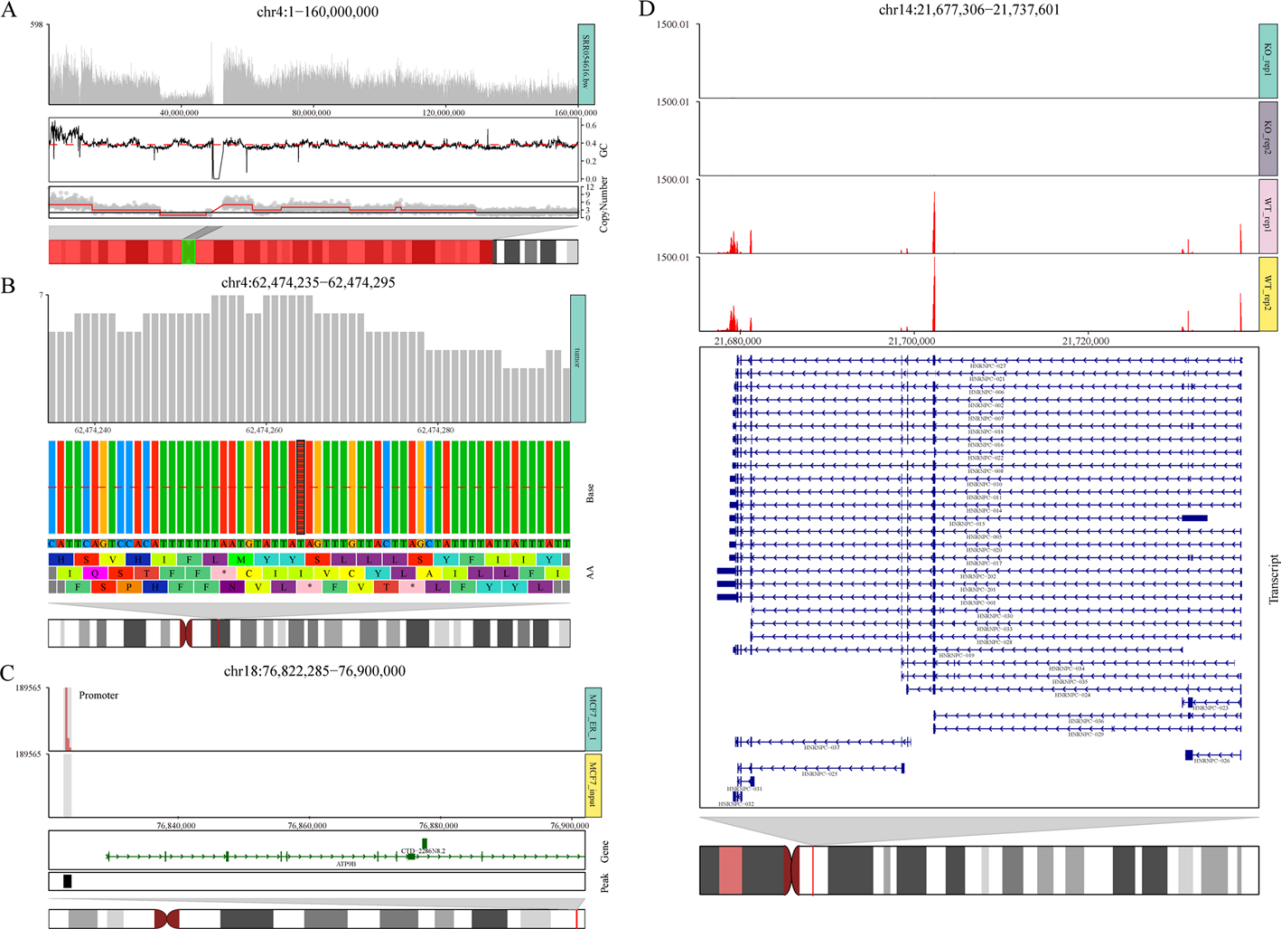
Visualizations of *ggcoverage* on selected NGS datasets. **A** *ggcoverage* on WGS data to visualize CNV. Genome coverage plot shows read counts of all bins. GC content annotation shows GC content of every bin (red line: mean GC content of the whole region). Copy number annotation shows normalized read counts of all bins in grey dot, estimated copy number in red line and ploidy in black line. Chromosome ideogram annotation shows the displayed region on the chromosome with red rectangle and highlights the centromeres with green rectangle. **B** *ggcoverage* on WGS data to visualize SNV. Genome coverage plot shows read counts of every locus. Base annotation shows base frequency (red line: 0.5) and reference base of every locus. Candidate SNV is highlighted with the twill mark. Amino acid annotation shows corresponding amino acids with 0, 1, 2 offsets. **C** *ggcoverage* on ChIP-seq data. Different from (**A**), genome coverage plot discriminates sample groups (the first track in red is the ChIP sample and the last track in grey is the control sample), the light grey rectangle indicates the highlight region. Gene annotation shows genes in given region (rightwards arrow with dark green: gene on plus strand, leftwards arrow with dark blue: gene on minus strand; element height: gene part (exon > UTR > intron)). Peak annotation shows all peaks identified. **D** *ggcoverage* on RNA-seq data with *HNRNPC* knockdown. Different from (**A**), we use transcript annotation instead of gene annotation to visualize gene’s all transcripts

Here, we show several practical use cases of applying *ggcoverage* to multi-omics, including WGS, ChIP-seq and RNA-seq. The code used to generate the figures without typesetting for Fig. 1 is available in Additional file 2.

In CNV analysis, common confounding factors include GC content bias, telomeres and centromeres proximity. Figure 1A shows genome coverage with all these confounding factors to inspect the data. When applying *ggcoverage* on WGS data to visualize SNV (Fig. 1B), we can see that there is a candidate single nucleotide variant (SNV) with T to A transversion at coordinate hg19 chr4:62,474,264 (highlight with twill), the variant allele frequency is 100%, and this may affect Y (Tyrosine), I (Isoleucine) and * (stop codon). When applying *ggcoverage* on ChIP-seq data (Fig. 1C), we can see that the ChIP sample has an enriched signal in the promoter region of the *ATP9B* gene compared to the input control, which is consistent with the results of called peaks. When applying *ggcoverage* on RNA-seq data with *HNRNPC* knockdown (Fig. 1D), we can see that there is a significant reduction in read coverage of *HNRNPC*.

## Conclusions

We have developed *ggcoverage*, an R package dedicated to visualizing and annotating genome coverage of multi-groups and multi-omics. It allows users to visualize genome coverage with flexible input file formats, and annotate the genome coverage with various annotations to meet the needs of different NGS data. In addition to visualization, *ggcoverage* also provides reliable and efficient ways to perform data preprocessing, including parallel reads normalization per bin, consensus peaks generation from replicates and track data loading by extracting subsets. And owing to the multi-platform support of R, users do not need to transmit data. Finally, it is very convenient to generate high-quality and publication-ready plots, users can also customize the figures with *ggplot2*.

## Availability and requirements

- Project name: *ggcoverage*
- Project home page: https://github.com/showteeth/ggcoverage
- Operating system(s): Unix, Linux, and Windows
- Programming language: R
- Other requirements: None
- License: MIT License
- Any restrictions to use by non-academics: None

### Supplementary Information

**Additional file 1. Additional figures. Fig. S1.** Three styles of genome coverage plot. **Fig. S2.** Highlight SNV with three styles. **Fig. S3.** Protein coverage plot based on peptides obtained by mass spectrometry and annotation of protein characteristics. **Fig. S4.** Examples of figure customization.

**Additional file 2.** The codes used to generate the figures without typesetting for Fig. 1.

## Data Availability

The datasets analyzed during the current study are available on GitHub (https://github.com/showteeth/ggcoverage).

## Abbreviations

NGS: Next-generation sequencing
WGS: Whole-genome sequencing
CNV: Copy number variations
RNA-seq: RNA-sequencing
ChIP-seq: Chromatin immunoprecipitation followed by sequencing
GTF: Gene transfer format
SNV: Single nucleotide variant

## References

[1] Nguyen D-Q, Webber C, Ponting CP (2006). Bias of selection on human copy-number variants. PLoS Genet.

[2] Conesa A, Madrigal P, Tarazona S, Gomez-Cabrero D, Cervera A, McPherson A (2016). A survey of best practices for RNA-seq data analysis. Genome Biol.

[3] Park PJ (2009). ChIP–seq: advantages and challenges of a maturing technology. Nat Rev Genet.

[4] Kent WJ, Sugnet CW, Furey TS, Roskin KM, Pringle TH, Zahler AM (2002). The human genome browser at UCSC. Genome Res.

[5] Robinson JT, Thorvaldsdóttir H, Winckler W, Guttman M, Lander ES, Getz G (2011). Integrative genomics viewer. Nat Biotechnol.

[6] Hahne F, Ivanek R. Visualizing genomic data using Gviz and bioconductor. In: Methods in molecular biology*.* 2016. pp. 335–51. 10.1007/978-1-4939-3578-9_16 27008022

[7] Yin T, Cook D, Lawrence M (2012). *ggbio*: an R package for extending the grammar of graphics for genomic data. Genome Biol.

[8] Skidmore ZL, Wagner AH, Lesurf R, Campbell KM, Kunisaki J, Griffith OL (2016). GenVisR: genomic visualizations in R. Bioinformatics.

[9] Gel B, Serra E (2017). karyoploteR: an R/Bioconductor package to plot customizable genomes displaying arbitrary data. Bioinformatics.

[10] Wickham H (2016). *ggplot2*: elegant graphics for data analysis.

[11] Ramírez F, Ryan DP, Grüning B, Bhardwaj V, Kilpert F, Richter AS (2016). deepTools2: a next generation web server for deep-sequencing data analysis. Nucleic Acids Res.

[12] Morgan M, Obenchain V, Lang M, Thompson R, Turaga N. BiocParallel: Bioconductor facilities for parallel evaluation. R package version 1.24.1. 2020.

[13] Jalili V, Matteucci M, Masseroli M, Morelli MJ (2015). Using combined evidence from replicates to evaluate ChIP-seq peaks. Bioinformatics.

[14] Pal K, Tagliaferri I, Livi CM, Ferrari F (2019). HiCBricks: building blocks for efficient handling of large Hi-C datasets. Bioinformatics.

[15] Pedersen TL. Patchwork: The composer of plots. R package version 1.0.0. 2020.

