# Supplementary material for: ggcoverage: an R package to visualize and annotate genome coverage for various NGS data
Source: BMC Bioinformatics. 2023 Aug 9;24:309. doi: 10.1186/s12859-023-05438-2 (PMC10413535; doi:10.1186/s12859-023-05438-2)

**Fig. S1.** Genome coverage plot with joint style (A), joint and group average style (B) and facet style with fixed Y-axis scale (C).


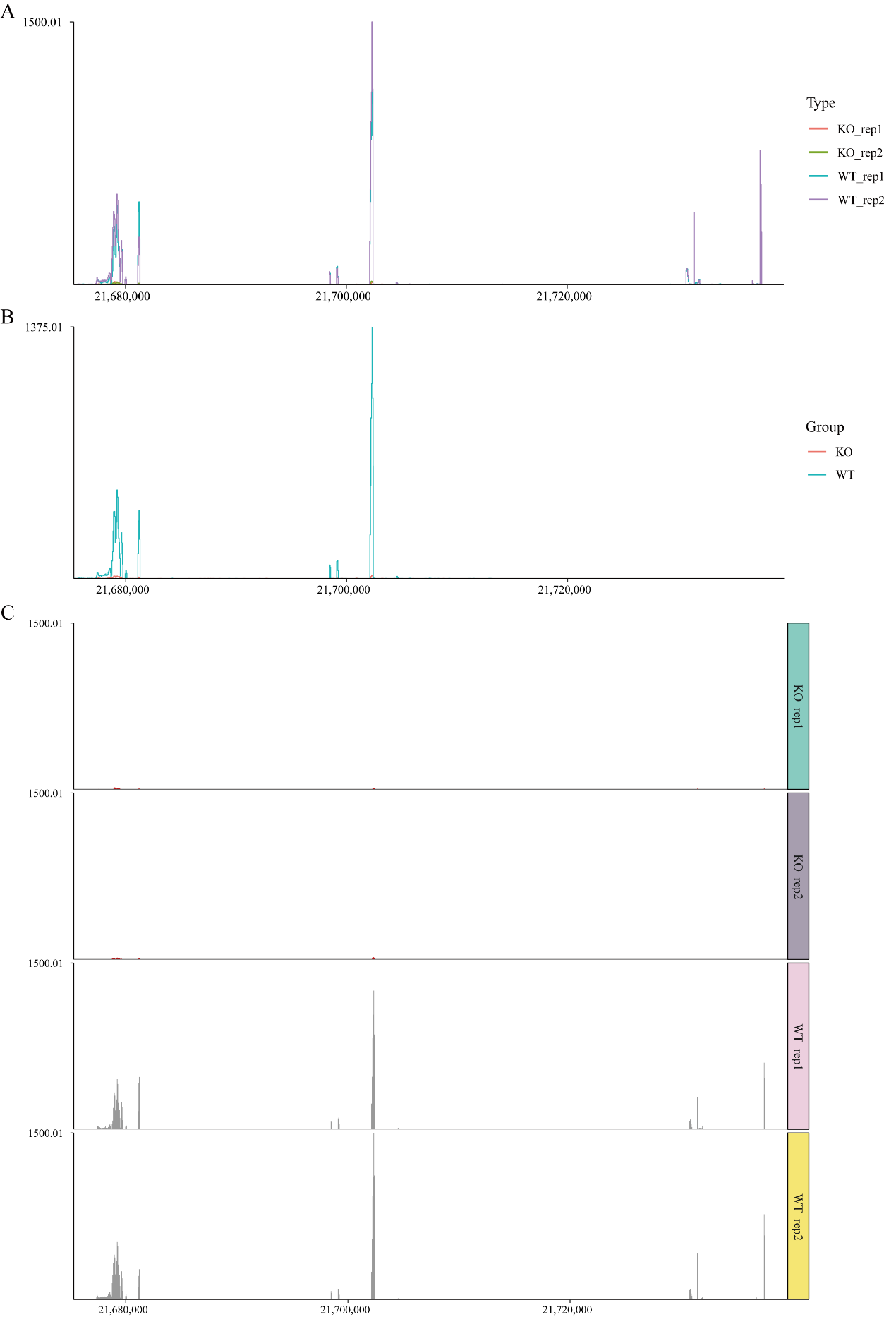


**Fig. S2.** Highlight SNV with twill (A), star (B) and color (C).


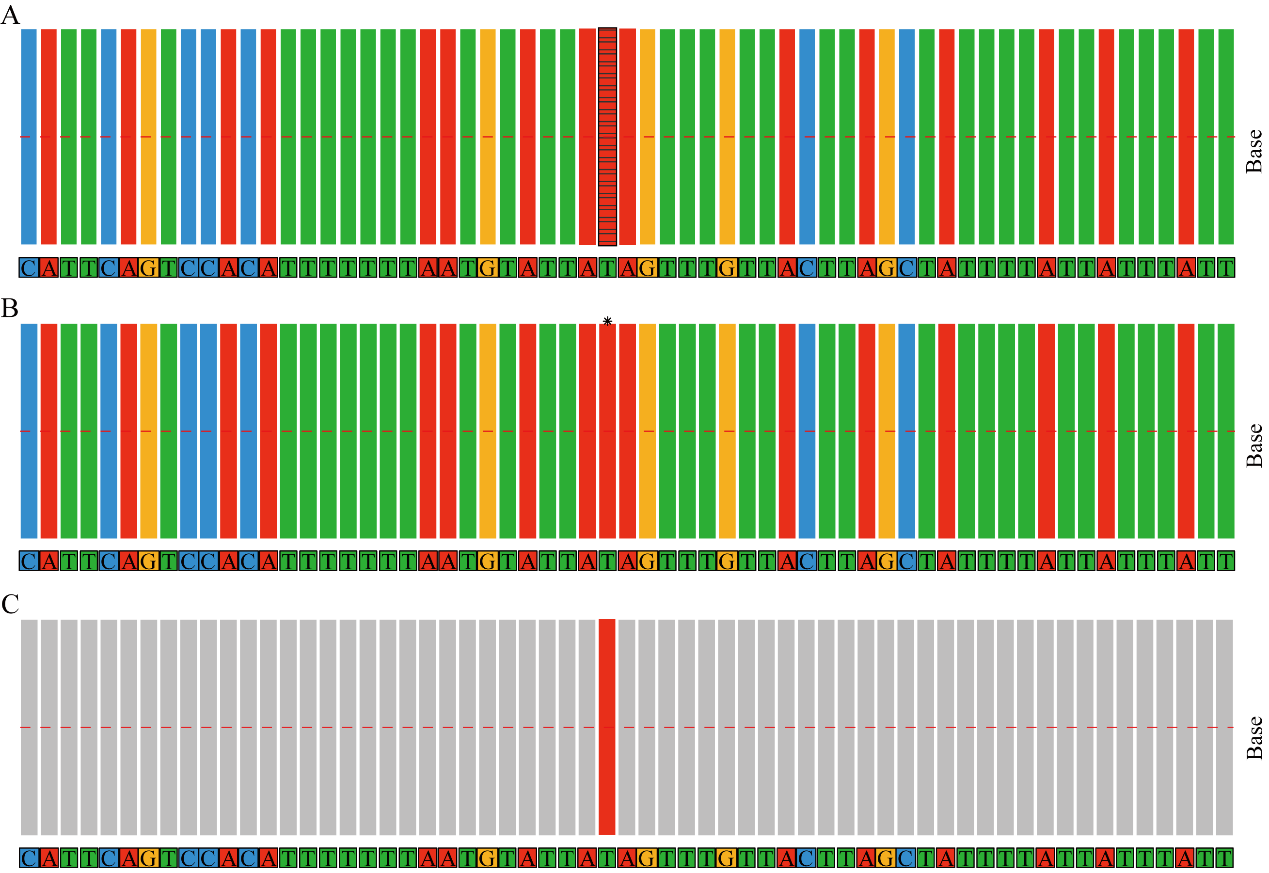


**Fig. S3.** Coverage plot of protein based on peptides obtained by mass spectrometry.


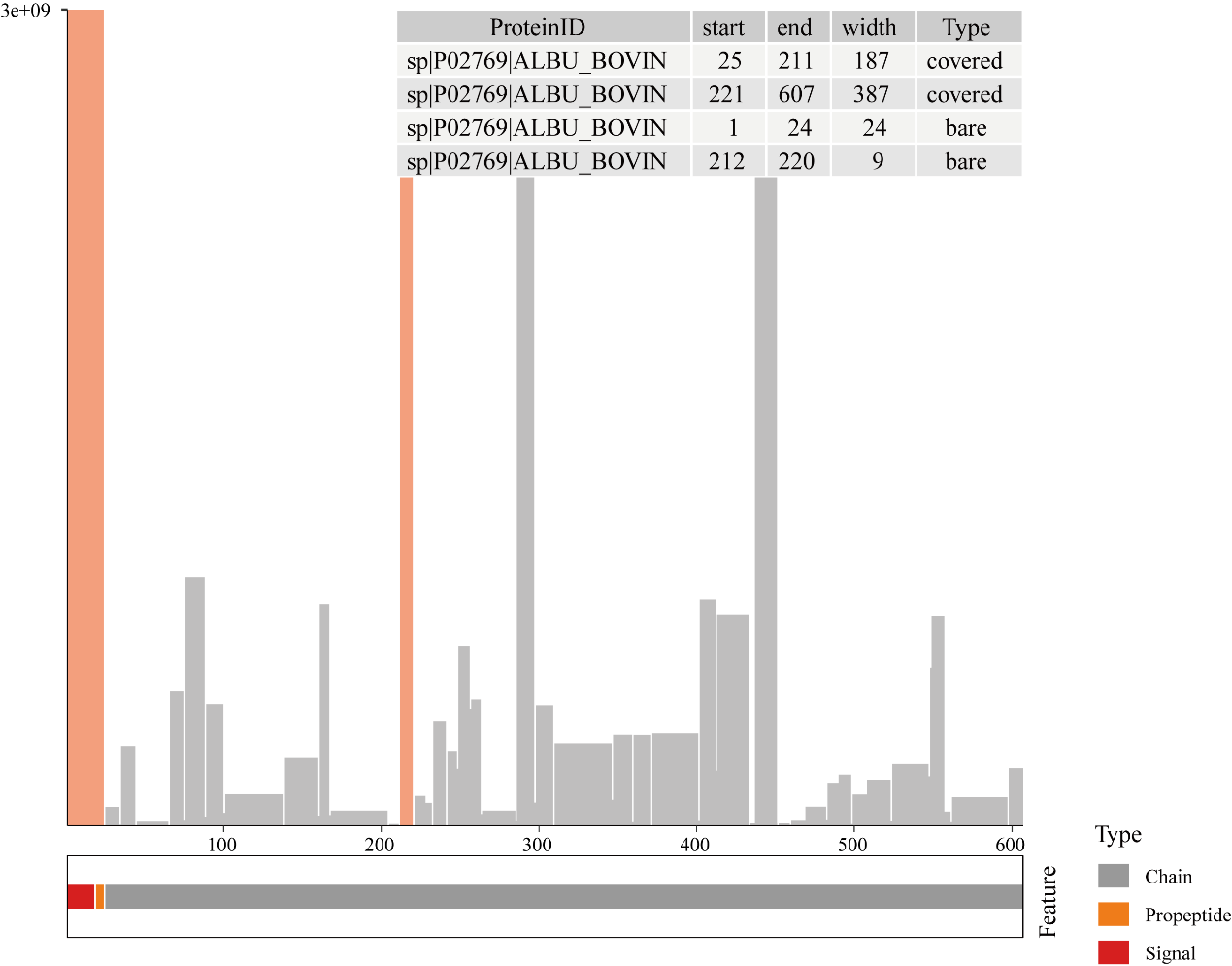


**Fig. S4.** Codes used to customize the figure elements (A), the output figure (B) and codes used to add a new layer (C), the output figure (D).


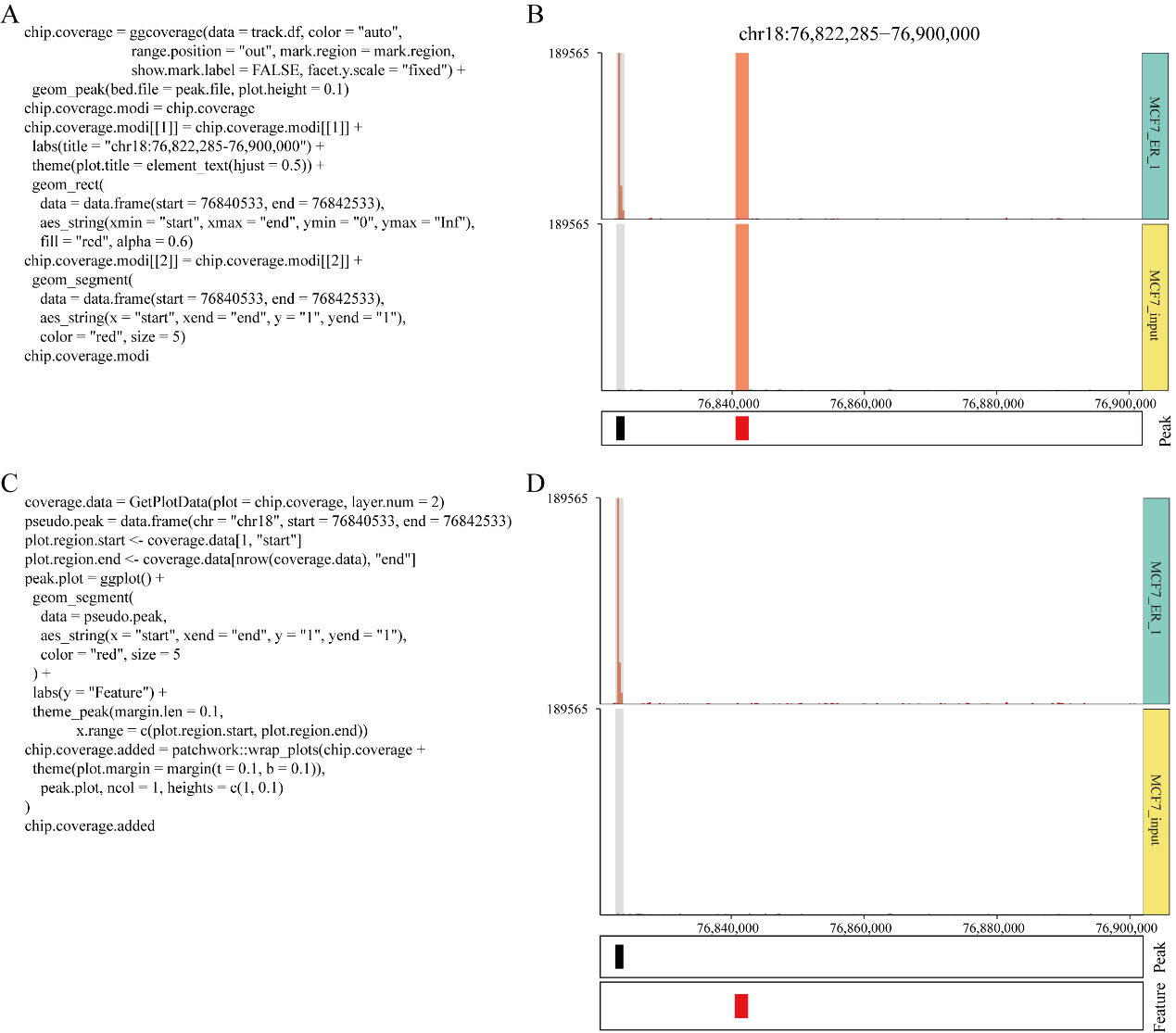

Supplement: Supplementary file 1 — Additional file 1. Additional figures. Fig. S1. Three styles of genome coverage plot. Fig. S2. Highlight SNV with three styles. Fig. S3. Protein coverage plot based on peptides obtained by mass spectrometry and annotation of protein characteristics. Fig. S4. Examples of figure customization. [file 12859_2023_5438_MOESM1_ESM.docx]
