# Supplementary material for: ggcoverage: an R package to visualize and annotate genome coverage for various NGS data
Source: BMC Bioinformatics. 2023 Aug 9;24:309. doi: 10.1186/s12859-023-05438-2 (PMC10413535; doi:10.1186/s12859-023-05438-2)
Supplement: Supplementary file 2 — Additional file 2. The codes used to generate the figures without typesetting for Fig. 1. [file 12859_2023_5438_MOESM2_ESM.pdf]

```
library(ggplot2)
library(tidyverse)
library(ggcoverage)
library(ggpattern)

meta.file <- system.file("extdata", "RNA-seq", "meta_info.csv", package = "ggcoverage")
sample.meta = read.csv(meta.file)
track.folder = system.file("extdata", "RNA-seq", package = "ggcoverage")
track.df = LoadTrackFile(track.folder = track.folder, format = "bw", meta.info = sample.meta)
gtf.file = system.file("extdata", "used_hg19.gtf", package = "ggcoverage")
gtf.gr = rtracklayer::import.gff(con = gtf.file, format = 'gtf')
basic.coverage = ggcoverage(data = track.df, color = c("grey", "grey", "red", "red"),
   mark.region = NULL, range.position = "out", facet.y.scale = "fixed")
RNA.coverage = basic.coverage + labs(title = "chr14:21,677,306-21,737,601") +
   theme(plot.title=element_text(hjust=0.5), strip.text = element_text(size=8)) +
   geom_transcript(gtf.gr=gtf.gr,label.vjust = 1.8, label.size = 1.6, arrow.length = 0.04,
      arrow.size=0.25, overlap.tx.gap = 0.2, tx.size = 0.75,
      utr.size = 1.5, exon.size = 2.5, plot.height = 1.5) +
   geom_ideogram(genome = "hg19", plot.space = 0)
ggsave(filename = "RNA_coverage.pdf",plot = RNA.coverage, width = 8,
   height = 11,dpi = 350, device='pdf')

sample.meta = data.frame(
  SampleName=c('Chr18_MCF7_ER_1', 'Chr18_MCF7_ER_2',
     'Chr18_MCF7_ER_3', 'Chr18_MCF7_input'),
  Type = c("MCF7_ER_1", "MCF7_ER_2", "MCF7_ER_3", "MCF7_input"),
  Group = c("IP", "IP", "IP", "Input"))
track.folder = system.file("extdata", "ChIP-seq", package = "ggcoverage")
track.df = LoadTrackFile(track.folder = track.folder, format = "bw",
  region = "chr18:76822285-76900000", meta.info = sample.meta)
track.df = track.df %>% dplyr::filter(Type %in% c("MCF7_ER_1", "MCF7_input"))
mark.region=data.frame(start=c(76822533), end=c(76823743), label=c("Promoter"))
basic.coverage = ggcoverage(data = track.df, color = "auto",
   range.position = "out", facet.y.scale = "fixed",
   mark.region=mark.region, show.mark.label = TRUE)
peak.file = system.file("extdata", "ChIP-seq", "consensus.peak", package = "ggcoverage")
chip.coverage = basic.coverage + labs(title = "chr18:76,822,285-76,900,000") +
   theme(plot.title=element_text(hjust=0.5)) +
   geom_gene(gtf.gr=gtf.gr, arrow.length = 0.04, arrow.size=0.25,
      gene.size = 0.75, utr.size = 1.5,
      exon.size = 2.5, label.size = 2.5, plot.height = 0.3) +
   geom_peak(bed.file = peak.file, plot.height = 0.1) +
   geom_ideogram(genome = "hg19", plot.space = 0, plot.height = 0.15)
ggsave(filename = "ChIP_coverage.pdf",plot = chip.coverage, width = 9,
   height = 4,dpi = 350, device='pdf')
```

```
track.file <- system.file("extdata", "DNA-seq", "SRR054616.bw", package = "ggcoverage")
track.df = LoadTrackFile(track.file = track.file, format = "bw", region = "4:1-160000000")
track.df$seqnames = paste0("chr", track.df$seqnames)
basic.coverage = ggcoverage(data = track.df, color = "grey",
   mark.region = NULL, range.position = "out")
cnv.file <- system.file("extdata", "DNA-seq", "SRR054616_copynumber.txt",
   package = "ggcoverage")
cnv.df = read.table(file = cnv.file, sep = "\t", header = TRUE)
library("BSgenome.Hsapiens.UCSC.hg19")
dna.coverage = basic.coverage + labs(title = "chr4:1-160,000,000") +
   theme(plot.title=element_text(hjust=0.5)) +
   geom_gc(bs.fa.seq=BSgenome.Hsapiens.UCSC.hg19, plot.height = 0.8) +
   geom_cnv(cnv.df = cnv.df, bin.col = 3, cn.col = 4) +
   geom_ideogram(genome = "hg19",plot.space = 0,
      highlight.centromere = TRUE, plot.height = 0.2)
ggsave(filename = "DNA_coverage.pdf",plot = dna.coverage, width = 9,
   height = 4,dpi = 350, device='pdf')

sample.meta <- data.frame(
   SampleName = c("tumorA.chr4.selected"),
   Type = c("tumor"),
   Group = c("tumor"))
bam.file = system.file("extdata", "DNA-seq", "tumorA.chr4.selected.bam",
   package = "ggcoverage")
track.df <- LoadTrackFile(
   track.file = bam.file, meta.info = sample.meta,
   single.nuc=TRUE, single.nuc.region="chr4:62474235-62474295")
snl.coverage = ggcoverage(data = track.df, color = "grey", range.position = "out",
      single.nuc=T, rect.color = "white") +
   labs(title = "chr4:62,474,235-62,474,295") +
   theme(plot.title=element_text(hjust=0.5)) +
   geom_base(bam.file = bam.file,
      bs.fa.seq = BSgenome.Hsapiens.UCSC.hg19,
      plot.space = 1, plot.height = 1.2, mark.type = "twill") +
   geom_ideogram(genome = "hg19",plot.space = 0,plot.height = 0.15)
ggsave(filename = "SNL_coverage_new.pdf",plot = snl.coverage, width = 9,
   height = 6,dpi = 350, device='pdf')
```
